# Clinical significance of circulating immune cells in left- and right-sided colon cancer

**DOI:** 10.7717/peerj.4153

**Published:** 2017-12-08

**Authors:** Jiabo Di, Meng Zhuang, Hong Yang, Beihai Jiang, Zaozao Wang, Xiangqian Su

**Affiliations:** Key Laboratory of Carcinogenesis and Translational Research (Ministry of Education), Department of Gastrointestinal Surgery IV, Peking University Cancer Hospital & Institute, Beijing, China

**Keywords:** Right-sided colon cancer, Circulating immune cells, Left-sided colon cancer, Lymph node metastasis prediction

## Abstract

**Background:**

Left-sided and right-sided colon cancers (LCCs and RCCs, respectively) differ in their epidemiology, pathogenesis, genetic and epigenetic alterations, molecular pathways and prognosis. Notably, immune response gene expression profiles have been shown to differ between patients with LCC and patients with RCC. The immune system plays an important role in tumor immunosurveillance, and there is increasing evidence that peripheral blood immune cells have a profound influence on tumor prognosis. This study aimed to determine the clinical significance of circulating immune cells with respect to colon tumor locations.

**Methods:**

Different types of circulating immune cells were separated and analysed based on their surface markers by flow cytometry. We compared the numbers of dendritic cells (DCs) and T cell subsets in the peripheral blood of 94 patients with RCC or LCC and analysed the proportions of these immune cells in relation to tumor stage, tumor differentiation and lymphatic metastasis.

**Results:**

We show that at later tumor stages, patients with LCC had higher levels of circulating myeloid DCs (*P* = 0.049) and plasmacytoid DCs (*P* = 0.018) than patients with RCC. In poorly differentiated tumors, LCC patients had significantly higher amount of plasmacytoid DCs (*P* = 0.036), CD4^+^ memory T (Tm) cells (*P* = 0.012), CD4^+^ T cells (*P* = 0.028), Tm cells (*P* = 0.014), and regulatory T cells (*P* = 0.001) than RCC patients. The levels of circulating CD4^+^ T cells, Tm cells and CD4^+^ Tm cells were significantly elevated at later stages in patients with LCC or RCC, while these cells decreased in poorly differentiated tumors in patients with RCC. Moreover, CD4^+^ Tm cell and CD4^+^ T cell levels are significantly associated with lymph node metastasis in patients with LCC and RCC.

**Discussion:**

Circulating immune cells were associated with tumor location, tumor stage and tumor differentiation, and can be used to predict lymphatic metastasis in patients with colon cancer. This variation in systemic immunity could contribute to the differential prognosis of patients with colon cancer.

## Introduction

Colorectal cancer is one of the five most commonly diagnosed cancers and the fifth leading cause of cancer death in China (Chen et al., 2016). Bufill (1990) first described the clinical disparities in incidence and outcome of colorectal cancer depending on the anatomical site of the primary tumor. Left-sided colon cancer (LCC) consists of cancers of the descending and sigmoid colon, whereas right-sided colon cancer (RCC) consists of cancers of the caecum and the ascending and transverse colon up to the splenic flexure (Iacopetta, 2002; Lee et al., 2015). Subsequent research has shown that tumor location influences the epidemiology, pathogenesis and prognosis of the disease (Iacopetta, 2002; Lee et al., 2015; Minoo et al., 2010). Right-sided lesions are more aggressive, which is reflected in tumor morphology and disease stage (Snaebjornsson et al., 2010). Patients with RCCs also have a significantly increased risk of locoregional recurrence (Park et al., 2015). Microsatellite instability (MSI) is an important molecular marker that indicates the pathophysiologic genesis of colon cancer, and provides some clinical information for treatment decision (Boland, 2007). Colon cancer with the MSI phenotype occurs more often in the right-sided colon (Takahashi et al., 2016). Notably, immune response gene expression profiles have been shown to differ between patients with LCC and patients with RCC (Minoo et al., 2010). These analyses further emphasize the distinct biology among colon cancers of different locations.

Cancer progression is a complex process involving multiple host—tumor interactions (Dunn, Old & Schreiber, 2004; Hanahan & Weinberg, 2011; Tosolini et al., 2011). The immune signature may predict tumor recurrence and patient survival in patients with all stages of colorectal cancer (Galon et al., 2006; Mlecnik et al., 2011). Besides local inflammatory responses, circulating immune cells also play an important role in tumor immune surveillance (Mohme, Riethdorf & Pantel, 2016). There is increasing evidence that circulating blood immune cells have a profound influence on tumor prognosis (Aliustaoglu et al., 2010; Letsch et al., 2000; Rana et al., 2015). Cancer cells that exit the primary tumor site—thus leaving the protection of the typically immunosuppressive tumor microenvironment—are more vulnerable to attack by immune effector cells (Mohme, Riethdorf & Pantel, 2016). These tumor cells need to escape destruction by circulating immune cells in order to seed a metastatic clone (Headley et al., 2016). However, thus far, the clinical significance of peripheral immune activity on LCC and RCC has not been determined.

During colon cancer metastasis, primary tumor cells may flow through the portal vein to the liver directly, or may migrate first to local lymph nodes and then distant lymph nodes and finally seed metastatic tumor in a distant organ, so that the extent of lymph node metastasis is a major determinant for the staging and the prognosis (Lindboe, 2011).  Moreover, tumor cell elimination by effector lymphocytes occurs in lymph nodes (Carriere et al., 2005). It remains unclear whether circulating immune cells can be used to determine the likelihood of lymphatic metastases.

The aim of this study was to investigate the clinical significance of the quantities of distinct types of circulating immune cells in patients with LCC or RCC. These cells included naïve T cells (Tn), memory T cells (Tm), regulatory T (Treg) cells, plasmacytoid dendritic cells (pDCs) and myeloid dendritic cells (mDCs). We show that circulating immune cells were associated with tumor location, tumor stage, tumor differentiation and lymphatic metastasis in patients with LCC or RCC.

## Materials and Methods

### Ethics statement

All procedures involving human participants were performed in accordance with the ethical standards of the research ethics committee of Peking University Cancer Hospital & Institute and with the 1964 Helsinki declaration and its later amendments (2015KT47). Written informed consent was obtained from all patients.

### Patient specimens

From 2015 to 2016, 94 colon cancer patients who underwent primary tumor resection at the Department of Gastrointestinal Surgery IV, Peking University Cancer Hospital were enrolled in this prospective study, and around 8 mL peripheral blood samples were obtained from each patient pre-operatively. Patients’ clinicopathological characteristics were determined according to the National Comprehensive Cancer Network (NCCN) guidelines for colon cancer (2016, Version 2).

### Microsatellite instability testing

For immunohistochemical staining, tissue slides (4 µm) were first deparaffinized in xylene, ethanol, and water, followed by treatment in 0.01 M citrate buffer (pH 6.0, 95 °C) for 10 min, and cooled down to RT. Slides were then steam treated (92−100 °C) in EDTA for 15 min. After cooling at RT and washing by PBS, to eliminate nonspecific binding of antibodies, endogenous peroxidase activity was quenched by immersing in 3% H_2_O_2_ for 15 min. The slides were then blocked with 5% milk for 1 h at RT. The sections were incubated with primary antibodies for MLH1, MSH2, MSH6 and PMS2 (1:500 diluted, Abcam, Cambridge, MA, USA) overnight at 4 °C, and then incubated with the corresponding secondary antibody labelled with HRP (1:250 diluted, Solarbio Life Sciences, Beijing, China) for 30 min at RT. The reaction products were visualized with DAB (Solarbio Life Sciences, Beijing, China) according to the manufacturer’s instructions, counterstained with hematoxylin, and differentiated by hydrochloric acid alcohol.

Microsatellite status was determined by two experienced pathologists according to immunohistological staining of the tumor tissue. Tumors expressing MLH1, MSH2, MSH6 and PMS2 were considered microsatellite stable (MSS) and patients lacking the expression of any of these markers were considered microsatellite instable (MSI) (Andersen et al., 2016).

### Isolation of peripheral blood mononuclear cells

To isolate peripheral blood mononuclear cells (PBMC) from the fresh blood samples, the blood was first diluted 1:1 with Iscove’s Modified Dulbecco’s Medium (IMDM containing GlutaMAX; Gibco-BRL, Paisley, Scotland), followed by density gradient centrifugation with Human PBMC isolation buffer (TBD Science, Tianjin, China) for 20 min at room temperature (RT). After separation, the PBMC layer was transferred to a new tube and washed with PBS (Thermo Fisher Scientific, Waltham, MA, USA) for 10 min at RT. Then cells were washed twice with Hanks (with calcium, magnesium, phenol red, Solarbio Life Sciences, Beijing, China) for 5 min at 4 °C and cryopreserved in liquid nitrogen.

### Cell labelling and flow cytometric analysis

To determine the cell phenotypes within the purified samples, we purchased anti-human CD1c-APC, CD127-APC, CD25-PE, CD45RO-FITC, CD123-FITC and CD303a-APC monoclonal antibodies from eBioscience (San Diego, CA, USA); anti-human CD45-PE, CD141-PE, CD11c-FITC, CD11c-PE-Cy7, CD8-PE, CD4 Alexa Fluor 488, CD4-APC and HLA-DR-PerCP-Cy5.5 antibodies from BD Pharmingen™ (San Diego, CA, USA); and anti-human CD16-BV421 and CD45RA-BV421 from BD Horizon™ (San Jose, CA, USA). To detect the various immune cells, PBMCs were stained with 3 µL of the above mentioned monoclonal antibodies for 30 min at 4 °C, washed twice in PBS containing 0.5% BSA and fixed in 1% paraformaldehyde (PFA, Sigma-Aldrich, St Louis, MO, USA). For quantification purpose, in each test 20000 PBMCs were counted by FACS.

### Quantification of circulating immune cells

**Figure 1:**
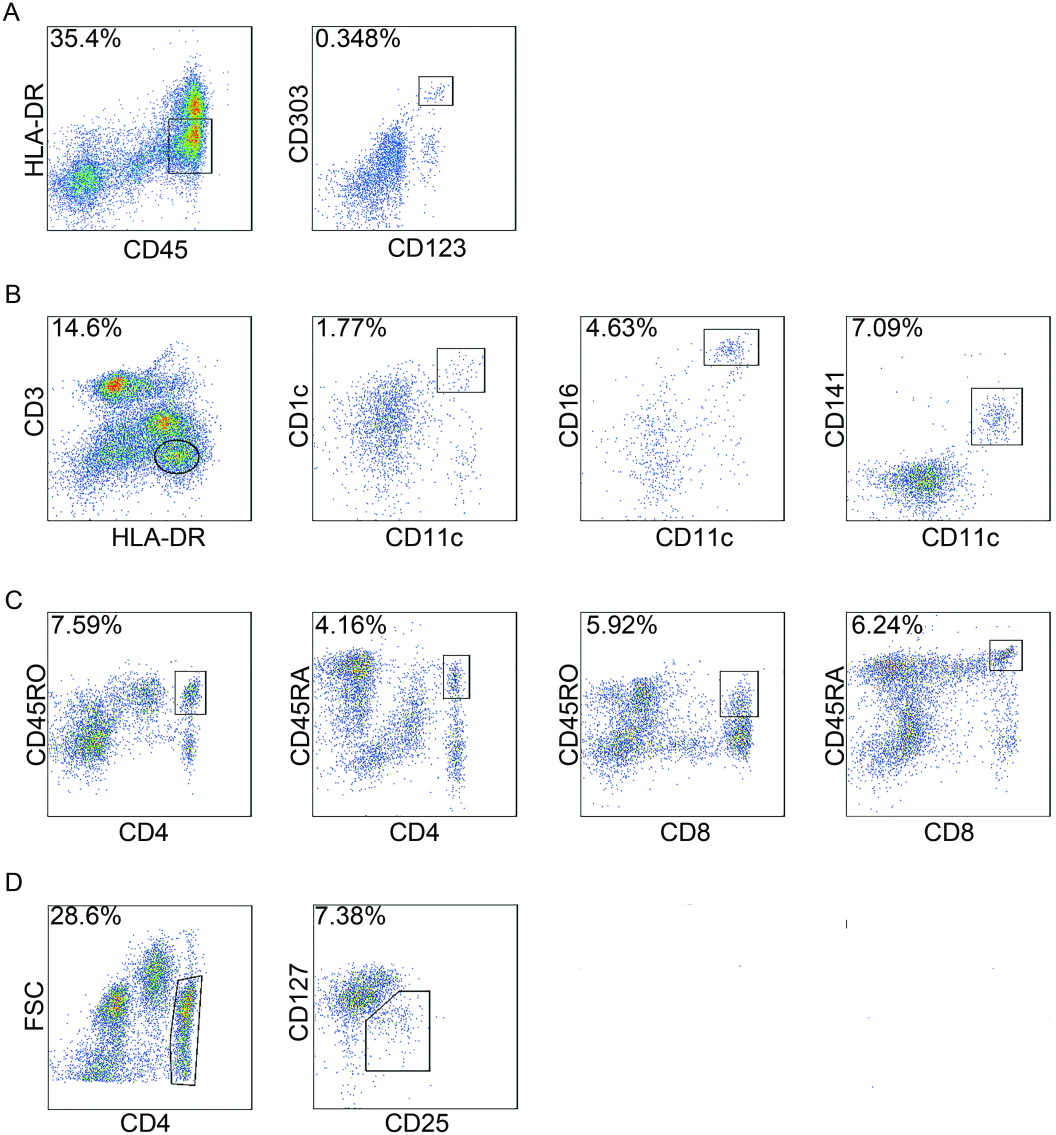
Gating strategies of circulating immune cells. Peripheral blood mononuclear cells (PBMCs) were isolated and analysed by flow cytometry. For each cell type, one patient was analysed just once, and 20,000 events were count for each sample by flow cytometry. In total, 94 patients were analyzed for circulating immune cells, and here the staining of a representative patient is shown to illustrate the gating strategy. (A) plasmacytoid dendritic cells (pDCs); (B) myeloid dendritic cells (mDC); (C) CD4^+^ naïve T cells (Tn) and memory T cells (Tm) , CD8^+^ Tn and Tm cells; and (D) regulatory T cells (Treg). FSC, forward scatter.

The number of cells in each subpopulation was measured using a BD FACS Aria SORP analyser (BD Biosciences, San Jose, CA, USA) equipped with 405, 488, 561 and 633 nm lasers, and the data were analysed with FlowJo version 7.6 (TreeStar, Ashland, OR, USA). The marker expression of different cells was determined by representative flow cytometry analysis in Fig. 1.

### Statistical analysis

Fisher’s Exact Test using R version 3.2.2 (R Foundation for Statistical Computing, Vienna, Austria, 2013) was performed to determine the correlations between clinicopathological features and tumor location. Student’s *t*-test (unpaired) was carried out using GraphPad Prism 5 software (GraphPad Prism Software Inc.; La Jolla, CA, USA) to examine the differences of circulating immune cells in patients with LCC or RCC, and results were shown as mean ± standard error of the mean (SEM). *P* < 0.05 was considered statistically significant.

## Results

### Patients with LCC or RCC have distinct clinicopathological characteristics

Of the 94 patients involved in this study, 55 had LCC and 39 had RCC. Patient clinicopathological parameters based on tumor location are shown in Table 1. Compared to patients with LCC, patients with RCC had significantly more poorly differentiated tumors (*P* = 0.002), more MSI tumors (*P* = 0.003), and larger tumors (*P* = 0.002). These data demonstrated that patients with LCC or RCC had different clinicopathological features.

**Table 1:** Clinicopathological characteristics of LCC and RCC patients.

Variables	LCC (%)	RCC (%)	*P*-value
**Age**			
<65 years	33 (60.00%)	30 (76.92%)	0.119
≥65 years	22 (40.00%)	9 (23.08%)	
**Gender**			
Female	24 (43.64%)	17 (43.59%)	1.000
Male	31 (56.36%)	22 (56.41%)	
**Depth of invasion**			
T1	2 (3.63%)	0	0.107
T2	11 (20.00%)	2 (5.13%)	
T3	35 (63.64%)	31 (79.49%)	
T4	7 (12.73%)	6 (15.38%)	
**Lymph node metastasis**			
N0	33 (60.00%)	25 (64.10%)	0.522
N1	15 (27.27%)	7 (17.95%)	
N2	7 (12.73%)	7 (17.95%)	
**TMN stage**			
I+II	32 (58.18%)	25 (64.10%)	0.669
III+IV	23 (41.82%)	14 (35.90%)	
**Tumor differentiation**			
Well/Moderately differentiated	49 (89.09%)	24 (61.54%)	**0.002**
Poorly differentiated	6 (10.91%)	15 (38.46%)	
**Histological subtypes**			
Adenocarcinoma	52 (94.55%)	32 (82.05%)	0.087
Mucinous carcinoma	3 (5.45%)	7 (17.95%)	
**MSI status**			
MSI	3 (5.45%)	10 (25.64%)	**0.003**
MSS	50 (90.91%)	24 (61.54%)	
Unknown	2 (3.64%)	5 (12.82%)	
**Tumor size** (*n*)	4.321 ± 0.2146 (53)	5.967 ± 0.5417 (36)	**0.002**

**Notes.**

LCC left-sided colon cancer RCC right-sided colon cancer T tumor burden *N* number of lymph node MSI microsatellite instable MSS microsatellite stable SEM standard error of the mean bold *P* < 0.05

### Correlation between circulating immune cells and tumor stages

pDCs are CD45^+^HLA-DR^low^CD123^+^CD303a^+^ (Piccioli et al., 2007; Tel et al., 2013). mDCs are CD3^−^HLA-DR^+^CD11c^+^ and can be further subdivided based on the expression of CD1c, CD16 and CD141 (Piccioli et al., 2007). CD45RA expression is indicative of Tn cells whereas CD45RO is a well-known surface marker that defines Tm cells (Sallusto et al., 1999). To assess the frequency of Tn and Tm cells in PBMCs from colon cancer patients, we quantified the CD4^+^CD45RA^+^ T cells, CD4^+^CD45RO^+^ T cells, CD8^+^CD45RA^+^ T cells and CD8^+^CD45RO^+^ T cells. Treg cells were determined by the presence of CD4^+^CD25^+^CD127^low^ (Venken et al., 2008) (Fig. 1).

To determine whether LCC and RCC patients have different levels of circulating immune cells, patients were divided into two groups according to their TNM stage—stages I + II and stages III + IV—as shown in Table 2. LCC patients in stage III + IV had significantly more CD16^+^ mDCs (*P* = 0.013) than RCC patients in stage III + IV. Moreover, LCC patients in stages III + IV showed a significantly higher quantity of total circulating mDCs and pDCs (*P* = 0.049 and 0.018, respectively) than RCC patient in stage III + IV.

**Table 2:** Circulating immune cells as a percentage of the total PBMCs at early and later tumor stages.

Cell type	Stage	Mean ± SEM of LCC (*n*)	Mean ± SEM of RCC (*n*)	*P*-value	95% CI
CD1c^+^ mDC	I + II	0.21 ± 0.03 (31)	0.28 ± 0.05 (25)	0.162	−0.17, 0.03
	III + IV	0.29 ± 0.06 (23)	0.14 ± 0.03 (14)	0.078	−0.02, 0.33
CD16^+^ mDC	I + II	0.63 ± 0.13 (31)	0.45 ± 0.080 (25)	0.280	−0.15, 0.50
	III + IV	0.74 ± 0.14 (23)	0.24 ± 0.06 (14)	**0.013**	0.11, 0.88
CD141^+^ mDC	I + II	1.20 ± 0.23 (31)	1.54 ± 0.57 (25)	0.549	−1.50, 0.81
	III + IV	1.55 ± 0.30 (23)	0.78 ± 0.35 (14)	0.115	−0.20, 1.73
mDC total	I + II	1.97 ± 0.35 (32)	2.27 ± 0.64 (25)	0.662	−1.70, 1.09
	III + IV	2.58 ± 0.48 (23)	1.16 ± 0.40 (14)	**0.049**	0.01, 2.84
pDC	I + II	0.29 ± 0.03 (30)	0.32 ± 0.04 (25)	0.651	−0.13, 0.08
	III + IV	0.32 ± 0.04 (22)	0.19 ± 0.03 (13)	**0.018**	0.03, 0.25
CD4^+^ Tm	I + II	6.69 ± 1.01 (31)	5.83 ± 0.82 (25)	0.522	−1.83, 3.56
	III + IV	11.82 ± 1.51 (22)	10.69 ± 2.18 (14)	0.662	−4.09, 6.35
CD4^+^ Tn	I + II	3.74 ± 0.63 (31)	4.18 ± 1.22 (25)	0.736	−3.05, 2.17
	III + IV	4.92 ± 0.73 (22)	5.86 ± 0.86 (14)	0.416	−3.27, 1.39
CD8^+^ Tm	I + II	3.93 ± 1.23 (31)	1.48 ± 0.36 (25)	0.088	−0.38, 5.28
	III + IV	5.40 ±1.18 (22)	2.42 ± 0.87 (14)	0.078	−0.36, 6.32
CD8^+^ Tn	I + II	5.46 ± 0.97 (31)	4.50 ± 0.86 (25)	0.475	−1.70, 3.61
	III + IV	4.80 ± 0.96 (22)	4.98 ± 1.15 (14)	0.906	−3.27, 2.91
CD4^+^ T cell	I + II	10.11 ± 1.45 (32)	10.01 ± 1.82 (25)	0.966	−4.51, 4.70
	III + IV	16.01 ± 2.02 (23)	16.55 ± 2.81 (14)	0.875	−7.42, 6.34
CD8^+^ T cell	I + II	9.09 ± 1.71 (32)	5.99 ± 1.00 (25)	0.150	−1.16, 7.37
	III + IV	9.76 ± 1.82 (23)	7.40 ± 1.36 (14)	0.366	−2.87, 7.58
Tm	I + II	10.29 ± 1.92 (32)	7.31 ± 1.00 (25)	0.209	−1.72, 7.68
	III + IV	16.47 ± 2.18 (23)	13.11 ± 2.88 (14)	0.355	−3.92, 10.64
Tn	I + II	8.91 ± 1.28 (32)	8.68 ± 1.67 (25)	0.914	−3.93, 4.38
	III + IV	9.30 ± 1.38 (23)	10.84 ± 1.39 (14)	0.461	−5.76, 2.67
Treg	I + II	1.49 ± 0.11 (32)	1.83 ± 0.52 (25)	0.473	−1.28, 0.60
	III + IV	1.72 ± 0.16 (22)	1.65 ± 0.17 (14)	0.775	−0.43, 0.57

**Notes.**

LCC left-sided colon cancer RCC right-sided colon cancer mDC myeloid dendritic cells pDC plasmacytoid dendritic cells SEM standard error of the mean Tm memory T cell Tn naive T cell Treg regulatory T cell CI confidence interval bold *P* < 0.05

**Figure 2:**
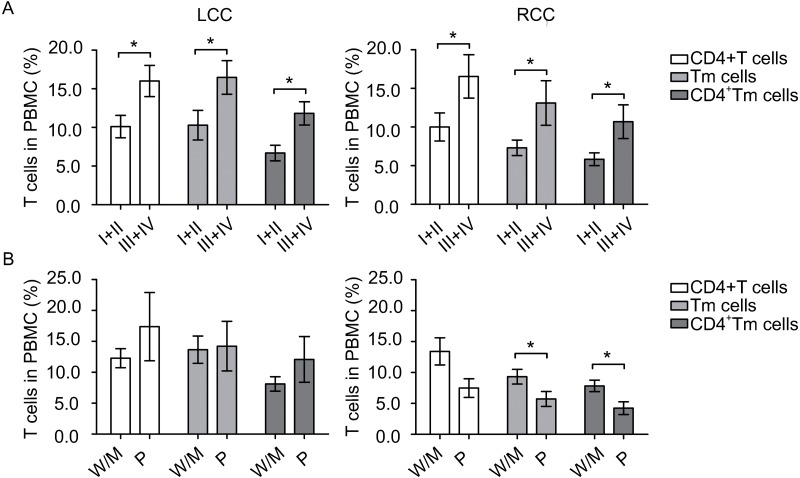
Circulating T cells are associated with tumor stage and tumor differentiation. The number of samples processed in CD4^+^ T cells, Tm cells and CD4^+^ Tm cells in Fig. 2A were the same as in Table 2, and that of Fig. 2B were the same as in Table 4. Statistical analysis was carried out using GraphPad Prism 5 software (GraphPad Software, La Jolla California USA) and unpaired Student’s t test was performed. (A) comparing T cells at different tumor stages in left-sided and right-sided colon cancer (LCC and RCC, respectively); (B) comparing T cells at different tumor differentiation status in LCC and RCC. ∗, *P* < 0.05. W/M, well/moderately differentiated; P, poorly differentiated.

To identify any potential relation between the amount of circulating immune cells and disease severity, we compared those cells at different tumor stages. Patients with early stage (I + II) tumors and patients with later stage (III + IV) tumors had similar amount of DCs. However, in LCC, patients with early stage tumors had significantly lower levels of CD4^+^ T cells (*P* = 0.018), Tm cells (*P* = 0.039) and CD4^+^ Tm cells (*P* = 0.005) than patients with late stage tumors (Fig. 2A). Similarly in RCC, patients with early stage tumors had significantly fewer CD4^+^ T cells (*P* = 0.049), Tm cells (*P* = 0.027) and CD4^+^ Tm cells (*P* = 0.018) than patients with late stage tumors (Fig. 2A). Collectively, these results suggest that the circulating DC levels differed between LCC and RCC patients, and circulating T cells levels were elevated during tumor progression to later stages.

### Circulating immune cells were correlated with tumor locations

In this cohort, the main population was patients with stage II or III diseases (*n* = 68). There were no significant differences in age, gender, TNM stage, and histological subtypes between stage II and III patients (Table S1). And no significant differences were observed between patients with stage II and stage III tumors in terms of DCs and T cell subsets (Table S2). Therefore, we considered that in patients with stage II and III colon cancer, tumor stage does not associate with the level of circulating immune cells.

Accordingly, we compared the circulating immune cells in patients with stage II and stage III colon cancer to investigate their relation with tumor location, and ruled out the effect by tumor stages. As shown in Table 3, our results show that patients with LCC had significantly more CD16^+^ mDC (*P* = 0.041), CD8^+^ Tm (*P* = 0.008), CD8^+^ T cells (*P* = 0.043), Tm cells (*P* = 0.011) and Treg cells (*P* = 0.009) than patients with RCC. These results indicate that circulating immune cell count was indeed associated with tumor location.

**Table 3:** Circulating immune cells as a percentage of the total PBMC in stage II/III LCC and RCC.

Cell type	Mean ± SEM of LCC (*n*)	Mean ± SEM of RCC (*n*)	*P*-value	95% CI
CD1c^+^mDC	0.24 ± 0.04 (36)	0.25 ± 0.04 (32)	0.831	−0.13, 0.10
CD16^+^ mDC	0.73 ± 0.13 (36)	0.41 ± 0.07 (32)	**0.041**	0.01, 0.62
CD141^+^ mDC	1.45 ± 0.25 (36)	1.44 ± 0.47 (32)	0.987	−1.02, 1.04
mDC total	2.42 ± 0.40 (36)	2.11 ± 0.53 (32)	0.635	−0.99, 1.62
pDC	0.32 ± 0.03 (34)	0.30 ± 0.04 (31)	0.631	−0.07, 0.12
CD4^+^ Tm	8.67 ± 1.13 (36)	6.37 ± 0.75 (32)	0.104	−0.49, 5.09
CD4^+^ Tn	4.32 ± 0.57 (36)	4.63 ± 0.98 (32)	0.778	−2.51, 1.89
CD8^+^ Tm	5.06 ± 1.20 (36)	1.49 ± 0.30 (32)	**0.008**	0.95, 6.19
CD8^+^ Tn	5.57 ± 0.85 (36)	5.04 ± 0.80 (32)	0.652	−1.81, 2.87
CD4^+^ T cell	12.99 ± 1.52 (36)	11.00 ± 1.51 (32)	0.359	−2.31, 6.30
CD8^+^ T cell	10.62 ± 1.69 (36)	6.53 ± 0.91 (32)	**0.043**	0.13, 8.07
Tm	13.73 ± 1.96 (36)	7.86 ± 0.90 (32)	**0.011**	1.38 , 10.37
Tn	9.89 ± 1.05 (36)	9.67 ± 1.41 (32)	0.900	−3.25, 3.68
Treg	1.67 ± 0.12 (36)	1.28 ± 0.07 (32)	**0.009**	0.10, 0.69

**Notes.**

LCC left-sided colon cancer RCC right-sided colon cancer mDC myeloid dendritic cells pDC plasmacytoid dendritic cells SEM standard error of the mean Tm memory T cell Tn naive T cell Treg regulatory T cell bold *P* < 0.05

### Circulating immune cells were associated with different differentiation status

It has been reported that patients with well differentiated colon cancer have a lower risk of lymph node metastasis than patients with poorly differentiated colon cancer (Derwinger et al., 2010). Moreover, patients with well/moderately differentiated colon cancer have a better prognosis than patients with poorly differentiated colon cancer. To understand whether this was associated to differences in the circulating immune cells, we analysed the circulating immune components of LCC and RCC patients based on the degree of tumor differentiation (Table 4). This analysis was also performed for patients with stage II and III diseases to reduce the effect by tumor stage. In patients with well/moderately differentiated tumors, patients with LCC had remarkably more CD8^+^Tm cells (*P* = 0.030) than patients with RCC. While in patients with poorly differentiated tumors, patients with LCC had significantly higher amount of pDCs (*P* = 0.036), CD4^+^ Tm cells (*P* = 0.012), CD4^+^ T cells (*P* = 0.028), Tm cells (*P* = 0.014) and Treg cells (*P* = 0.001) than patients with RCC. These results strongly suggest that in patients with poorly differentiated tumors, LCC patients had more circulating immune cells, which may indicate a better systemic immunity than RCC.

Moreover, to access whether circulating immune cells varies when the tumor differentiate to a more malignant status, we next compared immune cells at different differentiation grades. Patients with well/moderately differentiated tumors and patients with poorly differentiated tumors had similar amount of DCs. In LCC, patients with well/moderately differentiated or poorly differentiated tumors had similar amount of circulating T cells, as shown in Fig. 2B. In contrast, in RCC, patients with poorly differentiated tumors had borderline significantly less CD4^+^ T cells (*P* = 0.053), significantly less Tm cells (*P* = 0.048) and CD4^+^ Tm cells (*P* = 0.016, Fig. 2B). Taken together, these data suggest that tumor differentiation status was associated with circulating T cell levels in patients with RCC.

### Circulating immune cells are associated with lymph node metastasis

Lymph node metastasis requires breakdown of the host immune response in addition to escape of cancer cells from the tumor (Zuckerman et al., 2013). Therefore, we wondered whether the amount of different circulating immune cells was also correlated with lymph node metastasis in colon cancer. Indeed, we found that in RCC, patients without nodal metastasis had significantly more CD1c^+^ mDCs and pDCs than patients with nodal metastasis (*P* = 0.038 and *P* = 0.044, respectively, Table 5), while patients without nodal metastasis had significantly less CD4^+^ Tm cells, CD4^+^ T cells, and Tm cells (*P* = 0.018, *P* = 0.049, and *P* = 0.027, respectively) than patients with nodal metastasis. Moreover, in LCC, patients without nodal metastasis, CD4^+^ Tm cells and CD4^+^ T cells were also significantly lower than patients with nodal metastasis (*P* = 0.004 and *P* = 0.014, respectively, Table 5). These results indicate that the amount of certain circulating T cells increases whereas DCs decrease when lymph node metastasis occurs, and circulating CD4^+^ Tm and CD4^+^ T cell counts may serve as a predictor for lymph node metastasis in patients with colon cancer.

**Table 4:** Circulating immune cells as a percentage of the total PBMCs according to tumor differentiation status.

Cell type	Tumor differentiation	Mean ± SEM of LCC (*n*)	Mean ± SEM of RCC (*n*)	*P*-value	95% CI
CD1c^+^ mDC	W/M	0.24 ± 0.045 (31)	0.29 ± 0.054 (19)	0.434	−0.20, 0.09
	P	0.28 ± 0.12 (5)	0.20 ± 0.049 (13)	0.482	−0.15, 0.31
CD16^+^ mDC	W/M	0.79 ± 0.15 (31)	0.50 ± 0.098 (19)	0.161	−0.12, 0.70
	P	0.32 ± 0.10 (5)	0.28 ± 0.06 (13)	0.712	−0.20, 0.29
CD141^+^ mDC	W/M	1.56 ± 0.28 (31)	1.80 ± 0.75 (19)	0.722	−1.62, 1.13
	P	0.80 ± 0.35 (5)	0.92 ± 0.38 (13)	0.862	−1.50, 1.27
mDC total	W/M	2.59 ± 0.45 (31)	2.60 ± 0.83 (19)	0.989	−1.76, 1.73
	P	1.40 ± 0.57 (5)	1.40 ± 0.43 (13)	0.993	−1.66, 1.68
pDC	W/M	0.31 ± 0.034 (29)	0.33 ± 0.05 (19)	0.732	−0.14, 0.10
	P	0.41 ± 0.08 (5)	0.25 ± 0.03 (12)	**0.036**	0.01, 0.30
CD4^+^ Tm	W/M	8.12 ± 1.17 (31)	7.83 ± 0.92 (19)	0.861	−3.06, 3.65
	P	12.08 ± 3.70 (5)	4.23 ± 1.03 (13)	**0.012**	2.00 , 13.70
CD4^+^Tn	W/M	4.16 ± 0.58 (31)	5.58 ± 1.56 (19)	0.323	−4.27, 1.44
	P	5.30 ±2.13 (5)	3.25 ± 0.69 (13)	0.241	−1.52, 5.64
CD8^+^Tm	W/M	5.53 ± 1.38 (31)	1.49 ± 0.45 (19)	**0.030**	0.41, 7.66
	P	2.14 ± 0.62 (5)	1.48 ± 0.38 (13)	0.372	−0.86, 2.17
CD8^+^Tn	W/M	6.09 ± 0.95 (31)	5.10 ± 1.18 (19)	0.519	−2.07, 4.05
	P	2.32 ± 0.78 (5)	4.94 ± 1.00 (13)	0.144	−6.22, 0.99
CD4^+^T cell	W/M	12.28 ± 1.54 (31)	13.41 ± 2.20 (19)	0.669	−6.38, 4.13
	P	17.38 ± 5.52 (5)	7.48 ± 1.51 (13)	**0.028**	1.25, 18.57
CD8^+^T cell	W/M	11.62 ± 1.90 (31)	6.60 ± 1.37 (19)	0.064	−0.32, 10.36
	P	4.46 ± 1.37 (5)	6.42 ± 1.06 (13)	0.323	−6.03, 2.12
Tm	W/M	13.65 ± 2.21 (31)	9.32 ± 1.19 (19)	0.153	−1.67, 10.32
	P	14.22 ± 4.01 (5)	5.72 ± 1.20 (13)	**0.014**	1.99, 15.02
Tn	W/M	10.25 ± 1.15 (31)	10.68 ± 2.20 (19)	0.849	−4.97, 4.11
	P	7.63 ± 2.69 (5)	8.18 ± 1.30 (13)	0.837	−6.21, 5.09
Treg	W/M	1.59 ± 0.12 (31)	1.42 ± 0.08 (19)	0.331	−0.18, 0.52
	P	2.19 ± 0.38 (5)	1.07 ± 0.11 (13)	**0.001**	0.52, 1.72

**Notes.**

LCC left-sided colon cancer RCC right-sided colon cancer W/M well/moderately differentiated P poorly differentiated mDC myeloid dendritic cells pDC plasmacytoid dendritic cells SEM standard error of the mean Tm memory T cell Tn naive T cell Treg regulatory T cell CI confidence interval bold *P* < 0.05

**Table 5:** Circulating immune cells as a percentage of the total PBMCs according to lymph node metastasis.

Cell type	Tumor location	Mean ± SEM of N0 (*n*)	Mean ± SEM of N1 + N2 (*n*)	*P*-value	95% CI
CD1c^+^ mDC	LCC	0.20 ± 0.03 (32)	0.30 ± 0.07 (22)	0.105	−0.23, 0.02
	RCC	0.28 ± 0.05 (25)	0.14 ± 0.03 (14)	**0.038**	0.01, 0.27
CD16^+^ mDC	LCC	0.61 ± 0.13 (32)	0.77 ± 0.15 (22)	0.428	−0.55, 0.24
	RCC	0.45 ± 0.08 (25)	0.24 ± 0.06 (14)	0.074	−0.02, 0.45
CD141^+^ mDC	LCC	1.16 ± 0.23 (32)	1.61 ± 0.31 (22)	0.237	−1.20, 0.30
	RCC	1.54 ± 0.57 (25)	0.78 ± 0.35 (14)	0.356	−0.89, 2.41
mDC total	LCC	1.92 ± 0.35 (33)	2.68 ± 0.50 (22)	0.197	−1.95, 0.41
	RCC	2.27 ± 0.64 (25)	1.16 ± 0.40 (14)	0.230	−0.74, 2.97
pDC	LCC	0.29 ± 0.03 (31)	0.33 ± 0.04 (21)	0.517	−0.13, 0.07
	RCC	0.32 ± 0.04 (25)	0.19 ± 0.03 (13)	**0.044**	0, 0.26
CD4^+^ Tm	LCC	6.75 ± 0.98 (32)	11.98 ± 1.57 (21)	**0.004**	−8.75, −1.71
	RCC	5.83 ± 0.82 (25)	10.69 ± 2.18 (14)	**0.018**	−8.83, −0.90
CD4^+^Tn	LCC	3.69 ± 0.61 (32)	5.06 ± 0.75 (21)	0.164	−3.33, 0.58
	RCC	4.18 ± 1.22 (25)	5.86 ± 0.86 (14)	0.345	−5.25, 1.89
CD8^+^Tm	LCC	4.25 ±1.23 (32)	4.99 ± 1.16 (21)	0.678	−4.35, 2.85
	RCC	1.48 ± 0.36 (25)	2.42 ± 0.87 (14)	0.250	−2.57, 0.69
CD8^+^Tn	LCC	5.38 ± 0.94 (32)	4.89 ± 1.01 (21)	0.734	−2.36, 3.33
	RCC	4.50 ± 0.86 (25)	4.98 ± 1.15 (14)	0.741	−3.38, 2.43
CD4^+^ T cell	LCC	10.12 ± 1.41 (33)	16.26 ± 2.09 (22)	**0.014**	−11.01, −1.28
	RCC	10.01 ± 1.82 (25)	16.55 ± 2.81 (14)	**0.049**	−13.04, −0.04
CD8^+^ T cell	LCC	9.33 ± 1.67 (33)	9.43 ± 1.88 (22)	0.968	−5.24, 5.03
	RCC	5.99 ± 1.00 (25)	7.40 ± 1.36 (14)	0.405	−4.83, 2.00
Tm	LCC	10.66 ± 1.90 (33)	16.20 ± 2.26 (22)	0.067	−11.49, 0.42
	RCC	7.31 ± 1.00 (25)	13.11 ± 2.88 (14)	**0.027**	−10.91, −0.69
Tn	LCC	8.79 ± 1.25 (33)	9.50 ± 1.43 (22)	0.714	−4.57, 3.15
	RCC	8.68 ± 1.67 (25)	10.84 ± 1.39 (14)	0.388	−7.17, 2.85
Treg	LCC	1.48 ± 0.10 (33)	1.74 ± 0.17 (21)	0.177	−0.63, 0.12
	RCC	1.83 ± 0.52 (25)	1.65 ± 0.17 (14)	0.801	−1.25, 1.61

**Notes.**

LCC left-sided colon cancer RCC right-sided colon cancer mDC myeloid dendritic cells pDC plasmacytoid dendritic cells SEM standard error of the mean Tm memory T cell Tn naive T cell Treg regulatory T cells N lymph node CI confidence interval bold *P* < 0.05

## Discussion

This study focused on the quantification and comparison of circulating DCs and T cell subsets in patients with LCC or RCC, and we determined the clinical significance of immune cells with several measures of clinicopathological features. According to our data, patients with LCC may have better systemic immunity than patients with RCC, and these variations could be a response to the underlying tumor biology. Our results also indicated that circulating immune cells are associated with tumor location, tumor stage and tumor differentiation, and can be used to predict lymphatic metastasis, which may have potential indications for the future treatment of colon cancer patients.

The reason for the observed differences in circulating immune cells for patients with LCC and RCC remains unclear. We speculate that the difference in time to detection play a role. RCCs are often detected at later stages, due to weak symptoms. During tumor progression, the physical condition of patient becomes worse and worse, which may result in insufficient systemic immunity. Moreover, immune response gene expression profiles have been shown to differ between patients with LCC and patients with RCC (Minoo et al., 2010). Immunity in the tumor microenvironment may also affect the composition of circulating immune cells.

Our results show that RCC patients in the later stages of the disease have less circulating mDCs and pDCs than LCC patients; moreover, RCC patients in the later stages or with poorly differentiated tumors had fewer circulating DCs than patients in the early stages or with well/moderately differentiated tumors. In cancer patients, a decrease in circulating DCs might lead to abortion of T cell proliferation and antigen-specific responses, as well as unsuccessful cytokine production (Sakakura et al., 2006). Orsini et al. (2014) showed a significant reduction in the number of circulating pDCs in advanced-stage colorectal cancer patients compared to that in healthy controls. DC counts in peripheral blood are associated with prostate adenocarcinoma progression, and both pDCs and mDCs are significantly reduced in metastatic diseases (Sciarra et al., 2007). Therefore, our study may indicate a tumor progression-related immunosuppression in patients with RCC. Decreased level of DCs in high grade tumor suggest that these cells are likely to contribute in evaluating the degree of malignancy but follow up study is needed in order to draw any conclusion.

The present study suggests that circulating T cells were associated with colon cancer progression. Elevated levels of T cells were found in both right- and left-sided colon cancer patients with later tumor stages, whereas decreased levels of T cells were found in poorly differentiated tumors. In most cancers, the disease affected tissues generate tumor specific circulating immune cells. As the tumor grows, more tumor antigens were released which may increase the level of circulating Tm cells (Jane, Nerurkar & Karjodkar, 2007; Pages et al., 2005; Parcesepe et al., 2016). The Tm cells are important for mounting an immune response against the same tumor antigens if they are encountered again. However, RCC patients in the advanced stages or with poorly differentiated tumors consistently showed lower levels of T cells, indicating a less effective systemic immune response and could therefore also be a reason for the reduced survival rates of RCC patients.

The immunosuppressive function of Treg cells in colorectal cancer is still controversial (Salama et al., 2009; Tosolini et al., 2011). No difference between the prevalence of Treg cells and other cell populations was observed in either LCC or RCC patients, indicating the immune differences in LCC and RCC patients were not caused by differences in Treg cells. Similarly, Stanzer et al. (2008) detected an increased number of circulating Treg cells but also could not find a relationship between Treg and other non-Treg cells.

Lymph node metastasis is of great importance in treatment decision making in colon cancer. Just one nodal tumor deposit upstages the malignancy from N0 to N1. This is important as node-positive patients (N1) are considered for adjuvant chemotherapy whereas node-negative patients (N0) may not be (Sommariva et al., 2010). Current preoperative examinations cannot determine the nodal metastasis precisely, although TNM stage-based immunoscore has been recognized as a new component of cancer classification (Galon et al., 2014). Our results showed that circulating CD4^+^ Tm cells and CD4^+^ T cells might be indicators for lymph node metastasis, so that a liquid biopsy before surgery may provide useful node staging information for clinicians to make treatment decisions. Similarly in breast cancer, it was found that significant down-regulation of genes associated with immune-related pathways and up-regulation of genes associated with tumor-promoting pathways was consistently observed in the tumor, metastatic lymph node and blood immune cells, suggesting that such immune changes are not driven solely by local tumor invasion (Zuckerman et al., 2013).

There are still limitations in this study. Firstly, the study cohort is relatively small. Future studies with a larger cohort would help to enhance our findings. Moreover, functional studies are needed to unravel the relationship between circulating immune cells and survival of colon cancer patients with different tumor locations and to determine the underlying mechanisms that caused these differences.

## Conclusions

In conclusion, our results suggest that varying levels of immune cells were associated with tumor location, tumor stage, tumor differentiation and lymphatic metastasis in patients with colon cancer. These variations in systemic immunity could contribute to the differing prognosis of patients with colon cancer, and opens a new avenue for the management of patients with colon cancer.

##  Supplemental Information

10.7717/peerj.4153/supp-1 Data S1 Raw data

10.7717/peerj.4153/supp-2 Table S1 Clinicopathological characteristics of LCC and RCC patients in stage II/III

10.7717/peerj.4153/supp-3 Table S2 Comparing circulating immune cells in Stage II and Stage III patients

## Additional Information and Declarations

### Data Availability

The following information was supplied regarding data availability: The raw data has been provided as a Supplemental File.
