# Supplementary material for: Clinical significance of circulating immune cells in left- and right-sided colon cancer
Source: PeerJ. 2017 Dec 8;5:e4153. doi: 10.7717/peerj.4153 (PMC5724405; doi:10.7717/peerj.4153)
Supplement: Table S1 [file peerj-05-4153-s002.docx]

**Supplementary Table 1 Clinicopathological characteristics of LCC and RCC patients in stage II/III**

| **Variables** | **LCC (%)** | **RCC (%)** | **P-value** |
| --- | --- | --- | --- |
| **Age** |  |  | 0.595 |
| < 65 years | 24 (66.67) | 24 (75.00) |  |
| ≥65 years | 12 (33.33) | 8 (25.00) |  |
| **Gender** |  |  | 1.000 |
| Female | 16 (44.44) | 14 (43.75) |  |
| Male | 20 (55.56) | 18 (56.25) |  |
| **Depth of invasion** |  |  | 0.713 |
| T3 | 31 (86.11) | 29 (90.63) |  |
| T4 | 5 (13.89) | 3 (9.37) |  |
| **Lymph node metastasis** |  |  | 0.136 |
| N0 | 19 (52.78) | 23 (71.88) |  |
| N1+N2 | 17 (47.22) | 9 (28.12) |  |
| **TMN stage** |  |  | 0.136 |
| II | 19 (52.78) | 23 (71.88) |  |
| III | 17 (47.22) | 9 (28.12) |  |
| **Tumor differentiation** |  |  | **0.015** |
| Well/Moderately differentiated | 31 (86.11) | 19 (59.38) |  |
| Poorly differentiated | 5 (13.89) | 13 (40.62) |  |
| **Histogical subtypes** |  |  | 0.135 |
| Adenocarcinoma | 34 (94.44) | 26 (81.25) |  |
| Mucinous carcinoma | 2 (5.56) | 6 (18.75) |  |
| **MSI status** |  |  | **0.028** |
| MSI-High | 3 (8.33) | 10 (31.25) |  |
| MSI-low/MSS | 33 (91.67) | 18 (56.25) |  |
| Unknown | 0 | 4 (12.5) |  |
| **Tumor size (n)** | 4.731±0.2213 (36) | 6.300±0.6058 (31) | **0.012** |
| LCC, left-sided colon cancer; RCC, right-sided colon cancer; MSI, microsatellite instable; MSS, microsatellite stable; SEM, standard error of the mean; bold, P<0.05 | | | |
