# Supplementary material for: Clinical significance of circulating immune cells in left- and right-sided colon cancer
Source: PeerJ. 2017 Dec 8;5:e4153. doi: 10.7717/peerj.4153 (PMC5724405; doi:10.7717/peerj.4153)
Supplement: Table S2 [file peerj-05-4153-s003.docx]

| **Cell type** | **Mean ± SEM of Stage II (n)** | **Mean ± SEM of Stage III (n)** | **P-value** | **95% CI** |
| --- | --- | --- | --- | --- |
| CD1c^+^ mDC | 0.24±0.03 (42) | 0.26±0.06 (26) | 0.684 | -0.14, 0.09 |
| CD16^+^ mDC | 0.56±0.10 (42) | 0.61±0.13 (26) | 0.766 | -0.37, 0.27 |
| CD141^+^ mDC | 1.45±0.37 (42) | 1.45±0.31 (26) | 0.988 | -1.07, 1.05 |
| mDC total | 2.25±0.45 (42) | 2.33±0.46 (26) | 0.906 | -1.42, 1.26 |
| pDC | 0.31±0.03 (41) | 0.31 ±0.04 (24) | 0.925 | -0.09, 0.10 |
| CD4^+^ Tm | 6.51±0.76 (42) | 9.33±1.32 (26) | 0.051 | -5.66, 0.02 |
| CD4^+^ Tn | 4.04±0.78 (42) | 5.16±0.66 (26) | 0.320 | -3.36, 1.12 |
| CD8^+^ Tm | 3.31±0.94 (42) | 3.49±0.98 (26) | 0.900 | -3.01, 2.66 |
| CD8^+^ Tn | 5.35±0.74 (42) | 5.26±0.95 (26) | 0.938 | -2.31, 2.50 |
| CD4+ T cell | 10.55±1.32 (42) | 14.49±1.76 (26) | 0.074 | -8.29, 0.40 |
| CD8+ T cell | 8.66±1.31 (42) | 8.75±1.64 (26) | 0.968 | -4.29, 4.12 |
| Tm | 9.82±1.48 (42) | 12.82±1.90 (26) | 0.215 | -7.80, 1.79 |
| Tn | 9.39±1.15 (42) | 10.42±1.29 (26) | 0.565 | -4.58, 2.52 |
| Treg | 1.39±0.08 (42) | 1.64±0.15 (26) | 0.102 | -0.57, 0.05 |
| mDC,myeloid dendritic cells; pDC, plasmacytoid dendritic cells; SEM, standard error of the mean; Tm, memory T cell; Tn, naive T cell; Treg, regulatory T cell; CI, confidence interval. | | | | |

**Supplementary Table 2 Comparing circulating immune cells in Stage II and Stage III patients**
